# Children and adults rely on different heuristics for estimation of durations

**DOI:** 10.1038/s41598-023-27419-4

**Published:** 2023-01-19

**Authors:** Sandra Stojić, Vanja Topić, Zoltan Nadasdy

**Affiliations:** 1 Doctoral School of Psychology, ELTE Eötvös Loránd University, Budapest, Hungary; 2 Institute of Psychology, ELTE Eötvös Loránd University, Budapest, Hungary; 3 Faculty of Humanities and Social Sciences, University of Mostar, Mostar, Bosnia and Herzegovina; 4 Postgraduate Doctoral Study Program, Faculty of Humanities and Social Sciences, University of Zagreb, Zagreb, Croatia; 5 Department of Psychology, University of Texas at Austin, Austin, TX USA; 6 Zeto, Inc., Santa Clara, CA USA

**Keywords:** Human behaviour, Perception

## Abstract

Time is a uniquely human yet culturally ubiquitous concept acquired over childhood and provides an underlying dimension for episodic memory and estimating durations. Because time, unlike distance, lacks a sensory representation, we hypothesized that subjects at different ages attribute different meanings to it when comparing durations; pre-kindergarten children compare the density of events, while adults use the concept of observer-independent absolute time. We asked groups of pre-kindergarteners, school-age children, and adults to compare the durations of an "eventful" and "uneventful" video, both 1-minute long but durations unknown to subjects. In addition, participants were asked to express the durations of both videos non-verbally with simple hand gestures. Statistical analysis has revealed highly polarized temporal biases in each group, where pre-kindergarteners estimated the duration of the eventful video as "longer." In contrast, the school-age group of children and adults claimed the same about the uneventful video. The tendency to represent temporal durations with a horizontal hand gesture was evident among all three groups, with an increasing prevalence with age. These results support the hypothesis that pre-kindergarten-age children use heuristics to estimate time, and they convert from availability to sampling heuristics between pre-kindergarten and school age.

## Introduction

Time is a cardinal dimension we humans use to coordinate our social interactions and organize our memories and plans. Although a highly problematic concept in physics^1^, we perfected measuring it throughout history and mastered estimating it throughout our lifetime. Early development of temporal awareness, i.e., successful alignment with the temporal constraints, can be listed as one of the fundamental human skills. Refined "sense of time" leads to better adaptation to the environment^2^; therefore, early acquisition of the concept of time and a sense of it is desirable in kindergarten, critical in elementary school, and mandatory for personal success in professional life and our social interactions. Because time perception does not rely on dedicated sensory input, nor does it have a known neuronal representation like distance has^3,4^, there are ongoing philosophical debates and experimental work aiming to understand the neurobiological underpinnings of time tracking in the brain^5,6^ and children^7–11^ and the cognitive representation of time^12,13^.

The first and most overarching model of time estimation in humans and animals is the scalar expectancy theory (SET) by Gibbon^14,15^. Variants of this model are all based on a pacemaker assumption that an internal neuronal clock underlines human estimation of durations. Neuronal biophysical models differ in whether they attribute the pacemaker role to a central clock or distributed clocks in the brain^16^. This framework could explain dilation and compression of time perception by speeding up and slowing down the pacemakers by increasing the arousal^17–20^, by changing body temperature^21^, or by administration of drugs to control the level of dopamine in the brain^22–26^. In addition to these factors, an attentional modulation was also considered to contribute to time dilation by capturing the onset and the offset of the stimulus to be timed^27^. However, none of these models posit that time perception may rely on a two-layer biological and cognitive resource. The cognitive layer may be subject to age-related change that dramatically alters the method and outcome of time estimation. Because the estimation of durations does not rely on a dedicated feed-forward sensory system, but it is rather an indirect and inferential method, which, in the lack of an ideal central biological clock, satisfies Kahneman’s definition of heuristics: *“A heuristic is a mental shortcut that our brains use that allows us to make decisions quickly without having all the relevant information”*^28^.

Nevertheless, the results and their interpretation presented in this article are independent of the terminology of heuristics. At the cognitive layer, the time estimation heuristics reads out data from the neuronal layer that is most accessible for the individual to make a decision. We postulate that the mapping between biological and cognitive time may be subject to cognitive development; hence it changes by age. In this study, we investigated the effect of cognitive development on the estimation of durations.

There is no doubt we can discriminate durations, and this ability is based on a biological (pre-conceptual) level shared with animals. The evidence for duration discrimination has broadly been documented in various species. Animal species that can perform duration discrimination above chance level are pigeons^29^, rats^30^, cats^31^, monkeys^32^, dogs^33^. Likewise, temporal discrimination is present in infants and improves with age^34^. Tempo discrimination is also in place in infants 2–4 months old^35^. Based on all these findings from birds to infants, we have a good reason to assume that temporal discrimination at 5 years of age is well established. The ability to express temporal difference, however, is more than discrimination. It requires a cognitive representation of that interval. Since duration does not have a direct sensory trace, it requires a metarepresentation.

The other challenge of studying temporal discrimination in childhood is the uncertainty of mapping the duration in language. We cannot assert with certainty whether children of a particular age by using the word “duration” denote the same concept as an adult; i.e., the expression of duration such as “length” denotes the one-dimensional distance in time. The mere question “how long?” already implies a physical distance. Asking such questions may affect the conceptualization of answers. Therefore, inquiring about children’s cognitive representations by questions must be done carefully by not biasing their percept.

In physics, space and time, or space–time^36^, are intertwined. Disentangling space and time and defining their relationship, apart from physics, became a quest of cognitive scientists, such as conceptual metaphor theorists, and their idea of concrete over abstract domain mapping^37^. However, evidence of the psychological reality of spatial construal of time and critics from opponent theories are still a matter of discussion^38^.

Time–space dependency can also be apparent in people's integrated language systems– speech and gestures. That is, people talk about time with regard to space in spatial metaphors^39,40^ and produce spontaneous, or when required, deliberate gestures to depict temporal information spatially^41^.

One approach to eliminate the linguistic confound is using hand gestures instead of verbal answers. McNeill^42^ noticed that around the 3–4 years of age, there is an increase in gesturing, frequently referred to as a “gesture explosion,” that is abundant in so-called “silent gestures” or pantomiming iconic gestures and beat gestures^43^. However, the emergence of gestures that are metaphorical in nature occurs around to 5 or 6 years of age^44,45^. For instance, in metaphoric gestures, children appear to hold an object, as if objectifying what they are saying^44^, which is suggestive that children have the resources to produce metaphoric gestures for the time by this age and to represent an abstract content^43^. Further, it was proposed that those aspects of time that are not constrained by our physical experience with time are free to vary across languages, and our conceptions of them may be shaped by conventions, i.e., *"the way we choose to talk about them" *^46^. For instance, a study investigating a representation of duration and its orientation revealed that native English speakers tend to map the duration onto linear distance or horizontally (i.e., *long time*), while Spanish speakers map the duration onto quantity or vertically (i.e., *mucho tiempo*)^47^, same as Greek^47^, or Chinese^46^.

Regardless of whether the causes are linguistic structures or the easiness of a concrete domain over an abstract, or whether the Whorfian thesis of language relativity happens to be accurate to some extent, the question that imposes itself is: How do children reason about the temporal dimension before they adopt the metaphorical representation of time offered to them as a linguistic option? How does the estimation, navigation, and, subsequently, the conceptualization of time actualize before the concept of absolute time, typical of adults, is acquired? Is it about a single metric that becomes gradually differentiated, as proposed by Piaget^48^ (which would be congruent with contemporary Walsh's "A Theory of Magnitude"^49^, or whether children adopt the time–space relation during the pre-linguistic developmental period as a result of tracking useful cross-dimensional correlation^50^, and when would exactly the transition on the developmental timeline occur? In their paper from 2017, Magnani and Musetti^51^, for instance, have proposed that the metrical map of time was assumed to be innate, related to motor/implicit timing, and representing all the magnitudes with an analogue and bi-dimensional structure, while conceptual representation should be learned and related to cognitive-explicit time, offering an integrated and complementary approach.

Since time is neither being resolved by any sensory modality nor has an innate metric system to rely on, children may resourcefully apply heuristics. Heuristics are proxies utilized when direct representations are unavailable, e.g., when operating under various suboptimal conditions, such as time constraints, limited sources of information and cognitive capacities, or any other determining factors^52^. To act efficiently amidst such a restrictive milieu, various decision-making strategies are employed; instead of estimating the physical properties, the brain exploits invariants^53^. Such invariants, and in general, the concept of operating within "bounded rationality"^53^, was elaborated further by Kahneman and Tversky^54^ under the human decision-making models. They were the first to classify and elaborate on the heuristics of representativeness, anchoring, and availability. *“A person is said to employ the availability heuristic whenever it estimates frequency or probability by the ease with which instances or associations could be brought to mind”*^55^. *“Thus, a person could estimate the numerosity of a class, the likelihood of an event, or the frequency of co-occurrences by assessing the ease with which the relevant mental operation of retrieval, construction, or association can be carried out”*^55^, without the actual performance of the mentioned action. Another plausible concept, not necessarily heuristic by definition but rather its accompanying product, is sampling heuristic or sampling bias, where one accurately assesses the properties based on the samples of information available to them^56,57^. According to Fiedler and Juslin^58^, in such cases, participants behave as if they were naïve intuitive statisticians—summarizing the data accurately but being naïve about the potential biases in the data available to them^58^. Despite there is no consensus on how heuristics work, from Dennett's highly organized *intra-* and *inter-*systems relations of cognitive architecture^59^ to Gigerenzer's abstract and perplexed computational models^60,61^, no one can dispute their economic gain and effortless application at almost any age.

The aim of the present study was to observe whether different age groups conceptualize the time interval durations differently, both in verbal-based estimation and hand gestures. Because the shift from event-dependant to event-independent time, which resembles mature, abstract time, occurs around 5 years of age^62^, we hypothesized that the children before that age would conceptualize time according to the availability heuristic, using the content density of an interval given to estimate as a primary criterion and principle for conceptualization, i.e., *"how much they can talk about something"* rule, illustrated in a more children-appropriate narrative*.* The higher the perceived events' frequency, the more abundant the narratives, generating an impression of a longer duration. On the other hand, the adults were expected to rely on the sampling heuristics, based on the temporal particles of ordinary, everyday acts, that they could translate and tuck into the time window they were asked to estimate, following the *"how many times they could sample the absolute time"* rule. Furthermore, by asking the participants to gesture these durations, we wanted to gain an insight how they conceptualize the orientation of the timeline. Whether the magnitudes representing time are physically grounded on the vertical axis (implicating quantity) or the horizontal axis (representing length)? Because several studies pointed out that participants' non-linguistic duration estimates varied as predicted by the space–time metaphors in their native language^47^, we were curious to test how that would manifest in the Croatian language, which allows and uses both quantity and length representations. A simple Google search to quantify the prevalence of the two types of metaphors in Croatian did not leave us convinced to classify Croatian as a language with horizontal spatiotemporal metaphors, where the time is observed "as a distance," or vertical metaphor, where time is "quantity." Namely, an effortless search has revealed 15 million results for the expression *"puno vremena*," literally corresponding to the expression "*much time*" and 20 million results for the expression "*dugo vremena*," equivalent to English "*long time*" (Google, May 26, 2019, respectively).

Therefore, through analysing the chosen heuristic strategy underlying the duration estimation, an auxiliary question was asked: How may the orientation of hand gestures reflect these heuristics strategies as part of the integrated non-verbal language system?

**Figure 1:**
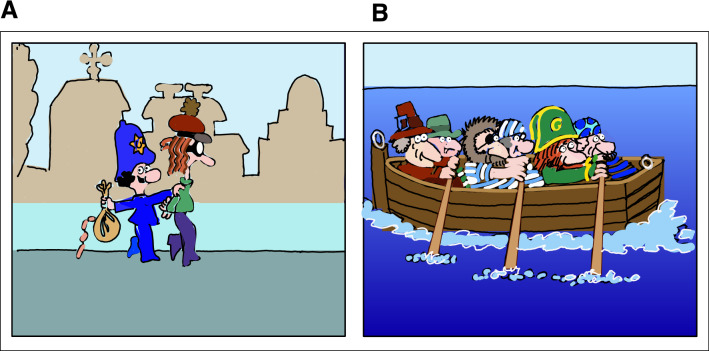
Screenshots from the cartoon videos: Image "(**A**)" on the left illustrates the action-packed video excerpt from an animated cartoon, accompanied by a rhythmically and melodically rich tune, while image "(**B**)" on the right illustrates another excerpt from the same animated cartoon but a rather monotonous and eventless sequence, with repetitive, and predictable actions, accompanied with a unison background music. The illustrations were redrawn from the original screenshots.

In the following experiment, we asked participants of three age groups, pre-kindergarteners, schoolers, and adults, to retrospectively compare the durations of two video clips, where video A was an eventful excerpt from a cartoon and video B was an uneventful counterpart of the same cartoon (Fig. 1). Unbeknown to the subjects, both clips were presented for equal durations in a balanced order across subjects. We hypothesized an interaction between the content- and age-dependency of the assertion of elapsed time.

We also asked our subjects to express the duration relations by spreading their arms without biasing them to use horizontal or vertical motions. The main variables of hypothesis testing were the binary tags of the videos (A, B) that appeared longer, the binary orientation of arm spreading, and the distance between arms expressing durations (See Methods).

We used three versions of the Chi-square test (Pearson’s Chi-square test, Likelihood Ratio, and Mantel–Haenszel test for trend) to determine whether the distribution of choices in duration comparison and arm spread orientation in our sample were homogeneous and independent of age. Having found an association between age and duration estimates, we tested whether the directions of differences were the same across all three age groups by expressing the pairwise differences in terms of odds ratios for each age group. In addition, we compared the arm distances across groups using a one-way ANOVA.

## Results

Verbal responses of N = 138 subjects' grouped in 3 age groups (n1_(4–5 YO)_ = 46, n2_(9–10 YO)_ = 46, n3_(>18 YO)_ = 46) were analysed after the exclusion of n = 2 subjects from the youngest age group because of the inconsistency between verbal estimation and arm spread. Since each subject's time estimate was nominal binary data, we quantified the responses as the frequency of choice A relative to B, where A was associated with "video A being perceived as longer than B" and B otherwise. We applied this quantification method consistently for all three groups. Chi-square tests of independence were used to test the statistical differences between A and B responses. Analytically, the hypothesis testing could be broken down into two null hypotheses: H0_A_ is to assume no difference in duration estimates within and across the three age groups relative to the uniform by-chance distribution; H0_B,_ contingent on the rejection of H0_A_, the response ratios of “video A was longer” relative to “video B was longer” are equal across all three age groups. Nevertheless, we combined the two tests into a single Chi-square test of independence. The contingency table of results is summarized in Table 1.

**Table 1:** The contingency table for the Chi-square test on the perceived duration of movie clips.

	Assertion of the video A longer	Assertion of the video B longer	*Row Totals*
Group 1 (4–5 YO)	31 (20.00) [6.05]	15 (26.00) [4.65]	46
Group 2 (9–10 YO)	18 (20.00) [0.20]	28 (26.00) [0.15]	46
Group 3 (> 18 YO)	11 (20.00) [4.05]	35 (26.00) [3.12]	46
Column totals	60	78	138 (Grand total)

Integer numbers represent the observed cell totals, numbers inside parentheses are the expected cell totals, and numbers in square brackets are the Chi-square statistic for each cell. Marginal distributions are indicated under Row Totals and Column Totals.

**Figure 2:**
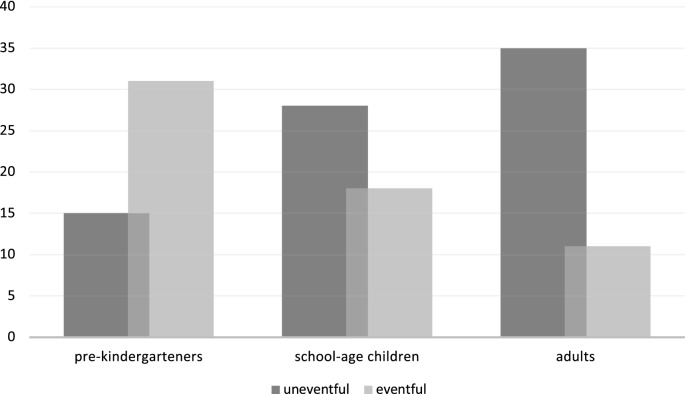
The number of subjects estimated one or the other type of videos as longer in duration by different age groups (pre-kindergarteners, school-age children, and adults).

First, we tested H0_A_ and examined the distribution of binary duration estimates across the three age groups (pre-kindergarteners, schoolers, and adults; Table 1). The contingency table of perceived durations showed highly polarized but very different response ratios across the three age groups confirmed by three independent statistics (P_Chi-sq_ < 0.001, P_L-ratio_ < 0.001; P_M-H test_ < 0.001); Table 2 and Fig. 2).

**Table 2:** Results of a Chi-square test of independence for different age groups (pre-kindergarteners, schoolers, and students) and type of video estimated as longer in duration.

	Value	Df	Asymptotic Significance (2-sided)
Pearson’s Chi-Square	18.223	2	0.000
Likelihood Ratio	18.683	2	0.000
Mantel–Haenszel test for trend	17.564	1	0.000
N of Valid Cases	138

Second, we tested H0_B_ and compared the imbalance in duration estimates between the three age groups by testing for uniformity vs. interaction. The between-group comparison of duration differences revealed a striking age effect. The direction of perceived duration differences of the two videos was the opposite between pre-kindergarteners and the two older groups (school-age and adults). A significant majority of pre-kindergarteners estimated the eventful video as longer than the eventless video, while school-age children and adults reported the opposite impression (χ^2^_(2, *N* = 138)_ = 18.22, *p* < 0.001). Accordingly, the odds ratio of adult subjects' judgment that the eventless video was longer than the eventful relative to the 4–5-year-old group’s preference was 6.5758 (*z* = 4.030, *p* = 0.0001), which is the same, due to the symmetry of logistic regression, as the odds ratio of pre-kindergarteners perceiving the eventless video as longer relative to the adult group’s assessment. The odds ratio of children in the pre-kindergarten group perceiving the eventful video as longer was significantly larger than in the school-age group (odds-ratio_(n=46)_ = 3.2148, *z* = 2.678, *p* = 0.0074). In contrast, the odds ratio of school-age children estimating the durations differently from the adult group was insignificant (odds ratio_(n=46)_ = 0.4889, *z* = 1.559, *p* = 0.1190).

In summary, the pre-kindergartener and adult age group estimated the duration of videos based on the event density with a robust age-wise interaction; that is, for pre-kindergarteners, the high action density was associated with a longer duration, while for the adults, the same high action density video was associated with a shorter perceived duration. Hence, the fact that the school-age group showed a pattern less polarized but statistically not different from the adult suggests a tipping point in cognitive development between pre-kindergarten-age and school-age when the concept of time and the heuristics applied to estimate durations fundamentally change (Fig. 2).

**Figure 3:**
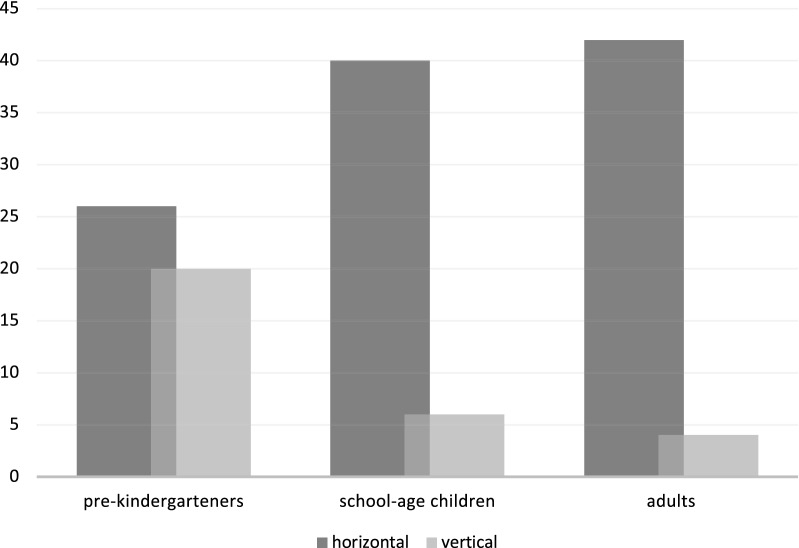
The direction of orientation in hand gestures in different age groups (pre-kindergarteners, school-age children, and adults). Dark and light shadings of bars represent horizontal and vertical arm spread expressions, respectively.

Lastly, we examined the orientation preference of hand gestures across the different age groups (pre-kindergarten, schoolers, and adults), which also revealed a significant age-dependent bias in hand gesture orientation Tables 3, 4/Fig. 3). As hypothesized, the predominance of representing duration with horizontal arm orientation was increasing with age, which we attribute to orthography and language. Again, the middle group demonstrated the same performance as the adults and strongly preferred to express the durations with horizontally oriented arm spreads (Fig. 3).

**Table 3:** The contingency table for the Chi-square test on arm-gesture orientation (the variables were explained in Table 1 caption).

	Horizontal arm gesture	Vertical arm gesture	Row totals
Group 1 (4–5 Y)	26 (36.00) [2.78]	20 (10.00) [10.00]	46
Group 2 (9–10 Y)	40 (36.00) [0.44]	6 (10.00) [1.60]	46
Group 3 (> 18 Y)	42 (36.00) [1.00]	4 (10.00) [3.60]	46
Column totals	108	30	138 (Grand total)

**Table 4:** Results of a Chi-square test of independence for different age groups (pre-kindergarten, schoolers, and adults) and the direction of orientation in hand gestures.

	Value	Df	Asymptotic Significance (2-sided)
Pearson’s Chi-Square	19.422	2	0.000
Likelihood Ratio	18.721	2	0.000
Mantel–Haenszel test for trend	16.237	1	0.000
N of Valid Cases	138

Despite the finding that in each group, participants tended to present the time through horizontal gestures (χ^2^ (2, N = 138) = 19.4222. *p* = 0.000061), when comparing the effect between age groups that tendency was expressed significantly more among the school-age children and adults than among pre-kindergarteners (school-age group: odds-ratio: 6.6667, z = 3.594, *p* = 0.0003; and adults odds-ratio = 10.5, z = 3.915, *p* = 0.0001). The large difference in the expression of time by horizontal versus vertical arm spreading was maintained from school-age to adults with no difference between the two age groups (χ^2^ (1, N = 92) = 0.4488, *p* = 0.5029). However, horizontal hand gestures’ predominance was no different from vertical in the pre-kindergarten age group (odds ratio_(N=46)_ = 1.300, z = 0.626, *p* = 0.5310).

Furthermore, we analysed the difference in arm spread length expressing the estimated duration of videos by a one-way ANOVA where age was the factor, and the distance between the arms (regardless of orientation) was the dependent variable. We did not find a significant age effect (*F* = 0.828, df = 2, *p* = 0.439, Table 5).

**Table 5:** Results of one-way ANOVA on the absolute differences of arm spread metric data between different age groups (pre-kindergarteners, schoolers, adults).

	SS	df	MS	F	p	η_p_^2^
Group	412.915	2	206.457	0.828	0.439	0.012

**Figure 4:**
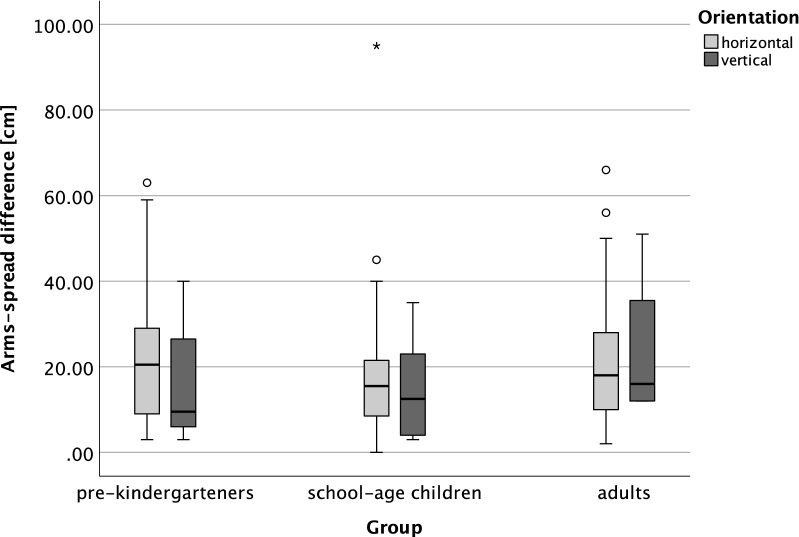
The box plot of arm spread differences in expression of relative durations, i.e., the difference between the expression of shorter and longer durations in centimeters, grouped by age groups (pre-kindergarteners, school-age children, and adults) and arm orientations. The lower and upper edges of gray rectangles represent the boundaries of the first and third quartiles of the data, respectively. The horizontal lines inside the boxes are the medians, and the whiskers represent the minima and maxima, excluding the outliers. Open circles and the asterisk represent outliers.

Lastly, we compared the variance of arm spread distances between the pre-kindergarteners and adults groups (Fig. 4) and observed no difference between the two age groups (Levene's test for equality of variance, *F* = 0.082, *p* = 0.775, *df* = 89).

## Discussion

The present study aimed to observe whether different age groups conceptualize the time intervals or durations differently, both in verbal and non-verbal expressions (hand gestures). Our underlying assumption was that the reference frame of duration estimates might change with age and education due to the increasing adherence to the concept of “absolute time” by adulthood, presumably starting at the first and second-grade education. In the absence of direct sensory representation of durations, we considered the choices of reference frames as heuristics. We hypothesized that pre-kindergarten-age children and adults use different heuristics to pivot answers to the question, “*Which one took longer?”* Hence, when it is a subject of comparison relative to another episode, the same episode may appear different in the time dimension. The intermediate school-age group was chosen as a middle point to reveal the trend of change and determine the likely age of the tipping point (Figs. 2 and 3). Our result that the school-age group (group 2) was found to take an intermediate position between the pre-kindergarteners (group 1) and adults (group 3) groups both in duration estimates and hand orientation confirmed the age-dependency of the conceptual change and placed the critical time window of switch between the age of 4 and 10.

To elucidate the fundamental difference in time perception between pre-kindergarten and adult ages, we used two animated short video clips of equal duration but different event densities. At the same time, the number of characters and moving elements were balanced. While the choice of the video was arbitrary, aside from the goal that both cuts had to be captivating for all three age groups, the subtle differences between the two videos at the level of physical features, in light of the results, did not matter, but we expand this argument below. One video was eventful (A), and the other was eventless, containing a lot of repetitive action (B) (Fig. 1). Our result suggests that both children's and adults' perception of time is influenced by the density of events, the number of episodes per time stored in the episodic memory, and the gaps between them. This event-oriented approach originated from a line of previous studies, which demonstrated that time, especially during a young age, is conceived as a quantitative dimension: *“any dimension on which different displays/events can be qualified as being more or less”*^63^; *“any more is more time”*^63^, *“time does not fly at a constant pace, its velocity depends on that of the event”*^48,63^, or Droit-Volet’s^64^ definition of a time as a *“quantity of information that has to be captured in time*.” Despite falling out of the scope of information-processing theories, i.e., cognitive models of time duration for longer durations, the event-oriented approach is not conflicted with one of the most prominent theories of psychological time, the scalar expectancy theory^14,15^, which among the others, represents the view that the time estimates are dependent on the number of pulses accumulated during the elapsed period, i.e., the more pulses are accumulated by the pacemaker, the longer the duration is perceived to be.

Despite the equal duration of videos, each age group ascertained the videos as different in duration, with a reversal of the difference between pre-kindergarten and school-age, which further increased in adults. We attribute this interaction to different heuristics underlying children's and adult's decisions: children, we posit, when estimating the duration of time intervals, were operating on the availability heuristic or *"how much they can talk about something"* rule, while the adults had a strong preference towards the sampling heuristic, or *"how many times they were able to sample the flow of absolute time" *rule.

When the subjects were instructed to represent the same magnitudes with simple hand gestures instead of verbally, pre-kindergarten children did not exhibit a preference for any orientation types and displayed both orientations with the same proportion. In contrast, school-age children and adults predominantly used the horizontal timeline to map temporal values. Explanation of why subjects of different ages exhibit such contrasting preferences, both in binary estimations and hand gestures, could be traced down to the developmental trajectories, both phylogenetically and ontogenetically, and inevitably affected by culture.

Various anthropological studies (e.g., performed on isolated cultures of Huni Kuĩ, Awetý, and Kamaiurá) reported that, along with reliance on diurnal and seasonal natural cycles, the content of a temporal interval was used to generate a sense of duration and give a structure to the non-metric time^65^. Such event-based time interval seems to differ largely from the concept used nowadays in Western culture, where, with late human contrivances, humans got accustomed to the artifacts of mechanical clock time and its product of uniform and abstract type of time^66^.

This transference of an "event-time" to "clock-time" pattern could be taken out of a broad picture and viewed ontogenetically; children who are developing the "sense of time" before being introduced to the conventional time should have relied on events as main building blocks to tell the time. In the early time-related developmental stages (3–7), events are utilized to reason about duration and later to help build a script-like order or decipher which occurred after another, i.e., before and later. That is also in line with a Piagetian framework of time development, who proposed that the preoperational children have a notion of time that is tied to events^48^ or that children's concept of temporal duration is not perceived but constructed based on inferential processes^67^. The extraction of events from the exterior and relying on content per se imposes availability heuristics as a natural choice of decision-making strategies in tasks requiring duration estimation. The ease of retrieval and construction generates a notion of abundance and fullness, making it appear longer, duration-wise.

To retrieve the content in a retrospective paradigm where the estimation request arrives unexpectedly, memory capacities have to be employed. Ornstein^68^ was one of the first to prioritize memory processes in the reconstruction of the event sequence and creating the sense of duration. In his “storage-size” hypothesis (1969), he addressed the effects of memory capacities, i.e., the limitation of memory storage and the accompanying complexity of the instances being stored, in a sense that, the greater complexity or higher number of stimuli are presented—the longer the perceived duration and the requested storage are.

Another memory-based model, the contextual-change model^69–71^, prioritized the changes in environmental, emotional, or other contextual elements. The more contextual changes are available for retrieval, the longer the perceived duration is i.e., *“the remembered duration is a cognitive construction based on the availability of contextual changes encoded in memory during the time period”*^72^.

Other information-processing models introduced attention as a relevant segment, e.g.,^73–75^ or elaborated the effects of retrospective and prospective methods of judgments^76,77^.

While “memory capacities” and “attention” play critical roles in discretizing and quantizing the information stored in memory, and they change with age, they should similarly affect the duration estimates in each age group. Hence, these factors do not explain the reversal of the duration estimation in school age and after. At no point in development, more information becomes less, and less information becomes more. If the quantization of content does not reverse with age, we reasoned, then the method of using the available information must change over time to infer durations from event quantities. This inferential process, i.e., the handling of information, is what we consider a type of heuristics without providing deeper insight into how the contents are organized and quantified in the brain of pre-kindergarteners and adults.

Arguably, “memory capacities” and “attention” may play key roles in time duration estimation and could explain why children would perceive several events to seem like “a lot” or, on the other hand, why the adults would not be affected exclusively by the quantitative aspect of the events. Due to the limited storage size^78^, and the dynamic nature of human attention^79^, it is highly unlikely that the pre-kindergarteners would register and retain all the relevant cues. Hence, for the 4–5-year-olds, the amount of retrievable content could serve as an indirect estimate of time. In the absence of an accessible and more accurate internal clock, we propose that the estimation of duration at that age relies on availability heuristics, i.e., the *“how much they can talk about something”* rule*.* This type of heuristics is most vulnerable to a content-dependent bias because the more non-repeating events occur in the mental storyboard of the video, the more they can talk about it. Therefore, if they can talk more about video A, then video A must represent a longer experience; hence it must capture a longer duration of time, consistent with our result.

In contrast, the sequence of events in those one-minute videos may not fill the memory storage capacities of adults. Hence, the unloaded memory capacity permits multitasking and accessing alternative sources of information indirectly linked to time, including but not limited to sampling the flow of “absolute time”, i.e., the Newtonian time^1^, or eventually, looking at a wristwatch. The potential bias deriving from this type of sampling heuristics is that the more opportunities the observer is given to sample the flow of absolute time, the more samples will be collected. Hence, when retrospectively comparing the durations of two experiences, one that was event-packed (video A) and the less eventful one (video B), the less eventful will allow for more sampling points of time. The sum of those sampling points amounts to the perceived duration; consistent with our result and explains the reversal of relative durations with age.

This model of age-dependent switching between heuristics posits that the concept of time becomes more abstract, less subjective, more action- and event-independent^62^, and as such, allocentric in its nature. The accessibility heuristics of time durations naturally evolves to sampling heuristics by age- and possibly by education, leading to acquiring the concept of a shared, absolute, and observer-independent time by the age of 10, which may eventually decline with aging and neurodegenerative diseases. Hence, concerning time perception, sampling heuristics may replace availability heuristics in parallel with the transition from egocentric to allocentric spatial reference frames about the age of 5.

Piaget also considered the relation between perceived duration and invested efforts. Accordingly, children estimate durations at a very young age as a function of the quantity of work accomplished or effort produced^48^. Specifically, an effort invested in tracking and cognitively processing the timeline of the events in the eventful video, when opposed to monotonous, repetitive, predictive, and therefore, less demanding, could result in perceiving the eventful video as longer in duration. This heuristic model of "event-time" that derives from the succession and frequency of the events should be abandoned once the concept of "clock-time" is adopted, not only declaratory but conceptually, together with progressive linguistic acquisition and use of metaphors. The "clock-time." i.e., a more externalized concept of time, where time itself would be perceived as *unidirectional*, *linear*, and *event-independent*^67^, would be subjected to a random sampling strategy as an underlying computing model. There, one could sample the external "absolute" time flow, aka. Newtonian time, by being aware of it and adding these "time awareness" moments together to account for the estimated duration. According to McCormack and Hoerl^62^, this shift from event-dependant to event-independent time, which resembles mature, abstract time, occurs around the age of 5.

Along with the transition from absolute to abstract time, *“the reduced sensitivity to time among younger children is primarily explained in terms of the more limited cognitive resources available to them, due to the development of attention and executive functions related to the slow maturation of the prefrontal cortex”*^64^. Young children, like animals, possess a fundamental mechanism that allows them to process time. However, the ability to judge time in different contexts heavily depends on supporting developmental functions^64^. Relative to school children and adults, a judgment of temporal duration among pre-kindergarteners might be primarily limited by the attentional span^80^ as well as the mechanisms of autobiographical and episodic memory and other timely occurring developmental trajectories related to the theory of mind and consciousness, concept introduction, language abstractiveness, and narrative flexibility or ability to read and write. Consequently, children are inclined to judge the temporal duration based on the non-temporal content^81^, and, therefore, are very sensitive to temporal illusions, such as empty-filled illusion or visual versus auditory illusion, both effects being greater in young children^82,83^. The improvements are not seen until the symbolic representation of time is acquired, around the age of 7, or until the adults’ general sensitivity to time is reached around the age of 8^64^.

For the same premises stated above, a contrasting outcome among different age groups was again expected in a subtask with hand gestures. Pre-kindergarteners were not expected to produce any homogenous pattern, *i.e*., display any preference towards the horizontal or vertical orientation of hand gestures (Tables 3, 4 and Fig. 3), as not yet being epistemologically immersed into the cultural products and inclined by their native language specificities and orthography. We intended to prove that children at that age are capable of processing the magnitudes (i.e., discriminating *short* form *long*, *big* or *small*) and, therefore, displaying arm spread with varying distances. A Theory of Magnitude (ATOM) suggests that the dimensions of space (i.e., size), time (i.e., duration), and number (i.e., quantity) are all processed by a single, innate magnitude processing system^84^. Hence, children at this age are able to discriminate differences in magnitude (for an elaborated discussion on children's understanding of "big "and "little, " see^85^) and answer whether something is *small-big* or *short-long.* The ability to discriminate magnitudes emerges early in the development, but it becomes more precise with increasing age, e.g.,^7,82^. Later this should crystallize into culturally inclined preferences, meaning that “*mental timeline and ATOM are not mutually exclusive* *theories*”^51,84,86^. Magnani and Musetti^51^, in a paper discussing the ontogenetic path of temporal reasoning, emphasized the existence of a metric and more advanced conceptual map, again suggesting that children up to a certain age do not appropriate to a mental-time line as a function to represent time. That being said, spreading arms could be learned from adults through conversational gesturing (of not necessarily temporal topics) and frequently asked questions such as "*show me how much you love me*,” "*how big is the sea*,” and "*how short is your younger brother,"* or *"how tall is your sand tower."* For instance, a response to parents' request "*show me how much you love me*" could be metonymically linked with the action of hugging as a visible part of loving someone and might be further generalized to denote a large magnitude. Nevertheless, in our study, even the youngest group showed consistency and expressed high confidence in their estimations when gesturing about duration, indicating an already established system for magnitudes and, therefore, a developing foundation for what would become a "sense of time." Only 2 children out of 48 were excluded, both 4-year-olds, for cardinal confusion (e.g., showing wider arm spread for a previously indicated video to end sooner). When representing the magnitudes of temporal durations, two other groups strongly preferred the horizontal representations exclusively, with a stronger effect visible among the adults (Tables 3, 4 and Fig. 3). Significant left-to-right bias strongly supports orthography's effects, i.e., reading/writing direction typical for Western culture, abundance and versatility of a particular language, and the general effects of education and exposure to Cartesian arrangements that we live in. Alongside the orientation, we analysed the exact distance between the arms in hand gestures. Those arm spread measurements served as an analogue response metric of the perceived magnitudes for both videos and helped us infer intra- and inter-age group variability and the relative ratio of two video durations. Intuitively, adults were expected to show less variability within the group; by using their internal reference, they might be competent to reason that the durations objectively did not differ significantly, regardless of their content. Furthermore, as we initially hypothesized, if operating on sampling heuristics, adults and adults-like respondents representing a duration as short as 1-minute were expected to display overall smaller arm spread when taking a relative stance compared to children. Accordingly, variability was expected to be higher among the children. However, a comparison of variances between the youngest and oldest age groups did not confirm that expectation. No difference in arm spread precision between age groups was found. We interpret the lack of effect as the variance of arm spread—in the schooler and adult groups was a combination of two opposing factors: the increasing consistency in estimation time intervals, and the random nature of the sampling heuristic. Some adults sample it more often than others resulting in different time assertions. The other factor compromising the consistency of arm spread responses might be attributed to the lack of reference. A potential future intervention could be done by introducing a fixed scale that would serve as a reference.

To complement current data, further studies addressing the heuristics as plausible underlying mechanisms for computing temporal properties could utilize the same concept but using a different experimental arrangement or another research approach.

Although the main difference between the two videos was the density of the events and the complexity of the narrative, we cannot rule out that the saliency of specific visual or acoustic features might have played a role in selectively capturing the attention of pre-kindergarten age children but not the adults’ and vice versa, hence could have potentially influenced the perception of durations. To address this point, we ran a control experiment.

In this follow-up study, we applied the same experimental conditions to the same age groups, except for using a different set of cartoons and, importantly, on this occasion, omitting the acoustic background. The silent version is particularly interesting because the relevance of the modality was extensively discussed in a series of previous works, both on duration filled and duration empty intervals^87–89^, demonstrating the prevalence of acoustics stimuli, with discrepancies being more pronounced during development than adulthood^83^. Nonetheless, the results were concordant with our original findings despite the absence of acoustic cues; hence the differential effect of auditory modality on children and adults does not account for the observed interaction in duration biases between age groups.

Besides the modal interaction, future variants could test for the effects of quantifiable contents, age-appropriateness, visual interest, musical style, number of characters, emotional tone, attention span and familiarity of the content and address whether the contrasting age effect is a result of maturation or the increased cognitive capacity of processing complex and intertwined story lines.

Furthermore, the frequency of "time-distance" and "time-quantity" metaphors in Croatian should be assessed experimentally instead of relying solely on approximate assessment and analyses^90^. Implicit vs. explicit in-depth analysis (e.g., spontaneous in-speech and deliberate temporal gestures), if dissociated, could assist us in pondering the relevance of a broader culture effect (direction of reading and writing) *vs.* language and language-embedded metaphors. Lastly, spatial and temporal awareness might be better approached when children are actively involved and performing actions rather than passively watching the presented content. Piaget^91^ has made the premise that a direct relationship exists between sensorimotor activity and spatial and temporal conceptualization. Young children appear to represent time better when it is coupled with a motor act^92,93^. Instead of the screened materials, short game-like scenarios with a matching amount of the events, ranging in complexities and content, could be arranged to implement such observations. In that sense, an egocentric frame of reference that is a natural stance appropriate for that age could be engaged^91,94^.

Our study left several aspects underlying the pre-kindergartener age concept of time yet to be investigated. Among those, the predictability of the chain of events, the familiarity of characters, the number of objects, animals, and humans, and their interactions and their roles in the study might have played an important role in parsing the story narrative. Other factors, such as the lack or presence of a story narrative, the conclusions or inconclusiveness of the story, the size of the vocabulary to verbalize the story, and the presence or absence of a listener to communicate the story to all may also influence the temporal embedding of events that manifest in the corpus of the content to estimate duration. All these questions are awaiting further investigation.

## Methods

### Participants

The final sample was composed of 138 participants, organized into 3-age groups. Sample sizes were aligned with previous studies concerning similar research interests^95^. Sex was balanced as equal numbers of girls and boys were admitted in each age group. The results of two participants were withdrawn (one girl, and one boy, both from the youngest group) as they did not seem to grasp the magnitudes and display consistency in their answers. The number of participants in the other two groups was set accordingly. The youngest group (*n* = 46, mean age = 4.7 years, *SD* = 0.59), enrolled at the state pre-kindergarten of Čapljina, Bosnia and Herzegovina, consisted of children with a wide range of socio-economic backgrounds. In order to take part in the study, the consent of parents or primary caretakers had to be provided in written form. The inclusion criteria were target age 4-year-olds and 5-year-olds with normal learning and communication abilities. Children with learning difficulties and developmental or neurological impairments were excluded. The same inclusion/exclusion criteria were applied to the second age group (*n* = 46, age between 9 and 10 years, mean age = 9.6, *SD* = 0.49) enrolled at the Primary School of the small town of Čapljina. The third age group, representing the adult sample (*n* = 46, mean age = 22.1, *SD* = 5.2), was enrolled at the University of Mostar, Faculty of Humanities and Social Science of Mostar. Participants were recruited electronically over the official announcement through the departmental mailing system. The subject enrollment was completed by signing the informed consent and verbally stating the absence of any endocrine, neurological, or psychiatric disorders and the use of any other modulators of an adrenal response or neuroactive substances. Participation in the study was entirely voluntary, without any means of compensation. Whether parents or children and students initiated it, participation was possible to quit at any time, without any consequences or explanation. Informed consents were obtained from all participants and their legal guardians. The study was conducted in compliance with the University of Mostar's research board rules and general rules of ethics defined by the "Declaration of Helsinki."

### Procedure

The study was performed in pre-kindergarten, elementary school, and an office at the University. Groups of pre-kindergarten and school children were tested in situ, while the adults were invited and tested individually at the Department of Psychology. All the participants were tested individually. During the experiment, participants were seated at the table. The task was presented with an LCD monitor of a PC (60 Hz refresh rate, 13-inch diagonal size) from a viewing distance of about 50 cm. All possible distractors were suppressed, and all time-tracking devices were removed from the examination room. All the testing was performed in the daytime and during the morning hours. The experiment consisted of two parts: (1) screening the videos and (2) the estimation part. Each participant was asked to indicate which of the two presented animated videos, A or B, subjectively appeared to be longer in duration. The binary forced-choice estimation was chosen as the easiest and simplest form of the perceptual assessment with the most consistent meaning across age groups. By providing “equal duration” as a third option, we would have had to explain the concept of “equality” to children, which could have complicated the task. In addition, the concept of “same” might have also introduced a confound as the “same duration” could be confused by the “same content” as the content and duration might not have been separated cognitively in our youngest age group of 4–5 years olds.

On the other hand, if any individual asserted the duration equal and made a by-chance forced decision, then at the group level, those individual by-chance decisions would manifest in the statistics as mixed responses. After verbal estimation, participants were asked to represent the durations of both videos with their hand gestures. Two measures were taken upon this act: (1) arm spread, i.e., the distance between the hands, that represented the perceived duration of videos, and (2) the orientation of hands (vertical or horizontal) that was used to infer the metaphorical conceptualization, i.e., whether the time was mapped as a "quantity" or as a "distance/length." The width of arm spread was measured with a tailor meter and expressed in centimeters, while the orientation was simply coded as "H" or" V" in the experimental log, the same as the "A" or "B" for binary estimates.

All the questions referred to the participants were carefully constructed, without any linguistic structures insinuating either the feature related to "length" or "quantity." Considering that Croatian allows metaphorically-dimorphic expressions when it comes to time conceptualization (e.g., *Koji crtić je bio duži?*—"Which cartoon appeared to last longer?"; *Koliko je trajao video?*—"How much lasted that video?" if using a literate, word-by-word translation), for all the participants, the task started with the neutral phrase "*Considering the duration, can you please compare cartoon A and cartoon B? How are they, duration-wise?*" (orig. “*S obzirom na trajanje, možete li usporediti crtani A sa crtanim B? Kakvi su?”*).

Despite the indirect question, children, including those in the youngest group, did not have a problem interpreting it. Those who, as a first attempt, offered adjectives that did not refer to the temporal aspect, such as “boring,” “interesting,” "good," etc., were encouraged to continue with their comparison *"Aha, okay. That is great! What else can we say about the cartoons? What are they like?* (Orig. “*Aha, u redu. Odlično! Što još možemo reći za crtiće, kakvi su?”*).

Each participant was verbally instructed to pay attention and observe carefully without revealing the main task or giving a more specific introduction. If the subject got distracted and looked elsewhere during a presentation, the experimentalist reminded them to refocus their gaze on the screen. The retrospective time estimation was applied, and each participant complied with the single-take estimation that occurred immediately after the screening set, including both videos. The order of presentation was pseudo-randomized across subjects such that half of the participants started the screening with video A and the other half with video B. The request to explicitly judge the duration of videos occurred without any previous notification or instruction. The time frame to provide an answer was not restricted; neither was a response time particularly pondered nor considered an additional dependent variable.

The experimenter manually logged all the answers to the log file. Both parts of the experiment, instruction and testing, lasted *cca.* 10 minutes per participant. The statistical analysis was performed using SPSS, IBM Statistics for Windows, Version 21.

### Task description

The videos were precisely the same in duration and were set to last for 60 s each but varied greatly in their content.

Regardless of their contrasting content, all sets were pleasant in valence, without disharmony in the acoustic domain, flickering and assailant images, or any uncomfortable actions in the visual domain. Both videos were presented on an anti-glare LCD laptop computer screen using a WLED backlight achieving 400-nits brightness at a 178° wide viewing angle and 60 Hz refresh rate. The horizontal x vertical video resolution was 960 × 720, respectively. The color palette, hue, luminance, and contrast were approximately equal between both videos. Likewise, the total motion energy and pixel change rate were equal, as evident from the file sizes (39.9 MB each) after MPEG-4 compression that uses discrete cosine transform as a compression method. While there was no difference in the graphical details and complexity between the two videos, the description of the two contents differed. The density of events was larger in video A than in video B, owning to the repetition and monotonicity in B and the lack thereof in A. Based on the stated properties and difference in event density among the experimenters for more straightforward nomenclature, the materials were referred to as an "eventful video" (or Video A) and "uneventful video" (or video B), respectively. Video materials were excerpts from "Professor Balthazar" (a "Zagreb film" production), Croatian animated series generated between 1967 and 1978. The “Zagreb film” production granted permission to use excerpts from the cartoons as the study’s main stimuli. All the edits, including the duration and manipulation of content, were performed in "OpenShot Video Editor" (OpenShot Studios, LLC), an open-source video editor. Both videos were novel to each participant, even if some of them might have been familiar with the cartoon series. Final videos, ready for individual assessments and comparison, were presented through a "PowerPoint presentation." Therefore, the outcome of the experiment was the datasheet with the list of durations—longer estimates given in a pair (of a pair A or B), coded along with the orientation preferences, and hand-indicated width for video A and video B (spreadsheet data as a CSV file is available at Supporting information, under Appendix 1).

### Statistical analysis

Our data was compiled into 2 × 3 contingency tables by (“A is longer”, “B is longer”) x (age groups 1–3) (Table 1, 3). To test the association between the age and binary duration estimates, we used three variants of Chi-square tests, all included in the SPSS software package: Pearson’s Chi-square test, Likelihood Ratio, Mantel–Haenszel test for trend (also called linear-by-linear test).

Pearson’s Chi-Square:

$${\chi }^{2}= \sum \frac{{({O}_{i }- {E}_{i})}^{2}}{{E}_{i}},$$

where χ^2^ is chi-square, *O* is the observed value, and *E* is the expected value.

The likelihood ratio is defined as:

$${\lambda }_{LR}=-2\mathrm{ln}\left[\frac{{sup}_{\theta \in {\Theta }_{0} }\mathcal{L}(\theta )}{{sup}_{\theta \in\Theta }\mathcal{L}(\theta )}\right],$$

where the expression represents the log-likelihood ratio of the parameter $\theta$ is in a specified subset ${\Theta }_{0}$ of $\Theta$, which is equivalent to the null hypothesis to test.

Mantel–Haenszel test (also called the Cochran–Mantel–Haenszel statistics):

$$\xi_{CMH} = \frac{{\left[ {\sum\nolimits_{i = 1}^{K} {\left( {A_{i} - \frac{{N_{1i} M_{1i} }}{{T_{i} }}} \right)} } \right]}}{{\sum\nolimits_{i = 1}^{K} {\left( {\frac{{N_{1i} N_{2i} M_{1i} M_{2i} }}{{T_{i}^{2} (T_{i} - 1)}}} \right)} }}^{2}.$$

The parameters are defined by the contingency table as follows:

**Table:** 

	Video A	Video B	Row total
Age group x	*Ai*	*Bi*	*N*_1*i*_
Age group y	*Ci*	*Di*	*N*_2*i*_
Column total	*M*_1*i*_	*M*_2*i*_	*T*_*i*_

A, B, C, and D cells represent the number of subjects stating that the specific video was longer than the other. The ${\xi }_{CMH}$ follows a Chi-square distribution asymptotically with 1 *df* under the null hypothesis.

These variants of the Chi-square test were applied to test the hypothesis if there was an association between age groups and duration estimates. In our case, these tests asserted a significant difference between the expected and observed choice ratios regarding the duration comparisons and arm spread orientations within each age group.

To compare the scalar variables of arm spread distances across different age groups, we used a generalized linear model one-way ANOVA.

### Ethics declaration concerning human subjects

The subjects’ participation was voluntary, and informed consent was obtained from all participants and their legal guardians. The study design has been approved by the ethical committee of the Faculty of Humanities and Social Sciences, University of Mostar, and performed in accordance with the Declaration of Helsinki. The study was performed following relevant guidelines and regulations.

## Supplementary Information

Supplementary Information.

## Data Availability

All the data supporting this study's findings are contained in Appendix 1. Explanation of column headers: “Code”: subject ID (S = school-age children, F = adult, V = pre-kindergartener); “Sex": (0 = female, 1 = male); “Age”: subject’s age in years; “Group”: age group (1 = pre-kindergartener, 2 = school-age children, 3 = adults); “Binary”: the choice of the video perceived as longer in duration (1 = eventless, 2 = eventful); “Orientation”: orientation of arm spread expressing the duration (0 = horizontal, 1 = vertical); “Simple”: arm spread distance expressing the duration of the eventless video in cm; “Complex”: arm spread distance expressing the duration of the eventful video in cm. “ratio”: the ratio of two arm spread distances; “difference”: the difference between the two arm spread in cm; “abs_difference”: the absolute value of the difference between the two arm spread in cm.
